# Understanding preferences for type 2 diabetes mellitus self-management support through a patient-centered approach: a 2-phase mixed-methods study

**DOI:** 10.1186/s12902-016-0122-x

**Published:** 2016-07-18

**Authors:** Janice M. S. Lopez, Bozena J. Katic, Marcy Fitz-Randolph, Richard A. Jackson, Wing Chow, C. Daniel Mullins

**Affiliations:** Janssen Scientific Affairs, LLC, 1000 US Route 202 South, Raritan, NJ 08869 USA; PatientsLikeMe Inc., 155 Second Street, Cambridge, MA 02141 USA; Harvard Medical School, 50 Milk Street, 17th floor, Boston, MA 02109 USA; University of Maryland School of Pharmacy, Saratoga Building, 12th Floor, 220 Arch Street, Baltimore, MD 21201 USA

**Keywords:** Type 2 diabetes mellitus, Diabetes management support, Patient participation

## Abstract

**Background:**

Patients with type 2 diabetes mellitus (T2DM) who participate in diabetes management programs have been shown to have better glycemic control and slower disease progression, although program participation remains low. In the USA, increasing participation in diabetes management support programs may also directly impact provider reimbursement, as payments are increasingly based on patient-centered measures. However, little is known about factors that may enhance patient participation. This study aimed at further understanding what is important in diabetes management support from the patients’ perspective and at assessing the utilization of various types of diabetes-management programs.

**Methods:**

A two-phase mixed-methods study was conducted of adult US members of PatientsLikeMe®, an online research network of patients. Phase 1 comprised qualitative interviews with 10 individuals to inform the online survey’s contents, aided by literature review. During phase 2, this online survey was completed by 294 participants who reported on their diabetes goals and preferences for T2DM self-management support programs.

**Results:**

The majority of the respondents were not participating in any program (65 %), but most had goals of improving diet (77 %), weight loss (71 %), and achieving stable blood glucose levels (71 %). Among those currently participating in programs, clinic, hospital-based, or other health-care professional programs were the most commonly used (51 %). The most preferred type of support was diet/weight-loss support (62 %), while doctors or nurses (61 %) and dietitians (55 %) were the most preferred sources of diabetes support.

**Conclusions:**

The low participation in diabetes self-management programs revealed in this study underscores the need for strategies to improve patient engagement. The results revealed support types and formats that patients with T2DM prefer and need. These findings may help improve patient engagement by guiding the future design of more effective diabetes management support programs.

**Electronic supplementary material:**

The online version of this article (doi:10.1186/s12902-016-0122-x) contains supplementary material, which is available to authorized users.

## Background

Type 2 diabetes mellitus (T2DM) affects approximately 29.1 million individuals in the USA and is primarily a self-managed disease in which self-care behaviors (e.g., following a healthy diet, being physically active, and taking prescribed medications) play a crucial role. It has been shown that engaging patients to take part in their own therapy can lead to quantifiable improvements in health-care quality and safety, particularly for chronic diseases such as T2DM [1, 2]. Patient engagement is increasingly being seen as a crucial component in the provision of high-quality health-care [3]. Joint guidelines published by the American Diabetes Association (ADA) and the European Association for the Study of Diabetes (EASD) recommend a patient-centered approach for the treatment of T2DM, in which patient preferences, needs, and values should be taken into consideration [4]. Furthermore, the Patient Protection and Affordable Care Act (PPACA) encourages patient-centered medical decision-making and greater patient engagement in the management of their own care [5]. Within the context of the PPACA, patient engagement is becoming an economic imperative as the Center for Medicare & Medicaid Services is increasingly shifting its reimbursement system to base payments upon quality measures surrounding patient outcomes.

As self-care behaviors play a crucial role in T2DM management, a better understanding is needed of the factors that influence patient engagement. A recent study found that a complex set of subjective experiential dimensions (cognitive, behavioral, and emotional) were involved in patient engagement, and it underscored the need to develop assessment tools to elucidate the nature of patient engagement from the viewpoint of the individual patient experience [6]. Perhaps unsurprisingly, it has been demonstrated that patients are more likely to adhere to treatment regimens that offer benefit from their own perspective [7, 8].

A range of diabetes management programs exists to help patients with T2DM monitor and manage their condition on a day-to-day basis and improve their outcomes. Although many diabetes-management programs have been shown to be beneficial for patients, formal program participation remains low [9, 10]. Little is known about what drives patient preferences for certain diabetes management programs above others and if patient characteristics play a role. As patient preferences, needs, and values increasingly become drivers of individualized treatment plans and of patient engagement, a clear understanding of the components of these elements could facilitate the design of better disease management programs that may result in improved patient participation, engagement, and adherence.

With the aim of improving future program implementation, Pimouguet et al. performed a meta-regression analysis of data from 41 randomized controlled trials to determine which elements of diabetes management programs are the most effective [11]. Two approaches emerged that were particularly effective: the ability for disease managers to modify the treatment of patients without prior physician approval, and a high frequency of patient contact. The authors concluded that the disease management approach resulted in significant improvements in glycemic control compared with conventional care [11].

The current study aimed to quantify and assess the utilization of various types of diabetes management programs among a real-world sample of patients with T2DM, in order to elucidate patient preferences for diabetes management and support. Following the Patient-Centered Research Outcomes Institute (PCORI) guidelines, patient-reported preferences and perspectives on support programming were first identified from a targeted subsample representing the T2DM patient population. These findings were then used to design and shape the study content [12].

## Methods

The study was conducted through PatientsLikeMe® (PLM) [13], an online platform comprising many disease communities where patients with life-changing medical conditions are able to find other patients like themselves, learn more about their condition, and share information about their outcomes. PLM has over 10,000 registered members with T2DM and approximately 8000 reporting T2DM as their primary condition, many of whom also report significant comorbidities. Patients with T2DM joining the website are asked to share information about their disease through custom questionnaires that populate their profile.

Following institutional review-board approval, a mixed-methods research process, including a comprehensive literature review, qualitative one-on-one patient interviews (January 2014), and a quantitative survey (April–July 2014), was used to comprehensively assess patient preferences regarding self-management support and diabetes management programs. Informed consent was obtained from all participants.

An extensive literature review of program support and assistance for patients with T2DM was first conducted to identify areas of evidentiary gaps for further exploration in the qualitative patient interviews. The areas identified included understanding the context in which patients make decisions about support for their diabetes (family structure, sources of assistance available), what the most important symptoms and problems requiring management are, who gives what kind of support, and which support programs patients may have tried in the past. Patients’ descriptions of their own goals, the relative importance of different kinds of support and support sources, and their preferences helped to inform the content of a structured survey, which was subsequently administered to a larger group of patients with T2DM.

### Qualitative interview process

A list was generated of PLM website users who reported T2DM in their patient profiles, had multiple log-in sessions including activity in diabetes forums, and were actively participating in discussions. Of the identified individuals, 44 were contacted, mainly by private message (using PLM email), and invited to participate in 1-h qualitative telephone interviews. Once 10 of the invitees had responded positively, no further invitations were sent out.

The respondents, half of whom were female, had a mean age of 57.5 years (range 34–78). Overall, 60.0 % (*n* = 6) were non-Hispanic white, and educational levels ranged from high-school diplomas to Master’s degrees.

Patients’ experiences with controlling blood glucose levels and managing symptoms and treatment regimens, and types of programs and support systems used were the key topics that guided the interviews. For a full description of the qualitative interview process, please see Additional file 1.

### Qualitative interview findings

Respondents reported common comorbidities including hypertension, hypercholesterolemia, depressive disorders, and chronic obstructive pulmonary disease. The majority perceived their blood glucose levels to be under control, although they were concerned about their weight and activity levels. Of the T2DM support systems/programs that respondents reported having participated in, most were one-time programs in which all follow-up was initiated by a nurse, not by the patient themselves.

Respondents reported that much self-education was performed through reading both online and printed materials. Websites commonly consulted included those of the ADA, PLM, insurance companies, and medical-supplies companies, as well as general medical websites. This was in agreement with information given by respondents that they preferred their materials to be from a ‘serious’ source and to be accessible at the patient’s own convenience. A few female respondents strongly favored participation in small groups for encouragement, sharing, and friendship; however, none actually belonged to such a group.

When questioned about their expectations/hopes for management programs, a range of responses were given, including the desire to not have diabetes or to slow or stop disease progression, to decrease or stop medication use, and to avoid complications (e.g., neuropathy, foot ulcers/amputation, heart disease, vision problems, peripheral artery disease). Respondents reported being motivated by wanting to keep learning and to avoid the negative consequences of their disease. They reported being discouraged from participating in programs because of the difficulty in navigating certain health-care systems and from continued participation due to repetition of material.

When asked from whom they received help in the management of their condition and what form that help took, responses included spouse, adult children, other family members (who often also had diabetes themselves), and online friends. Support and encouragement from health-care professionals (HCPs) was mostly related to diet and medication; additional encouragement and praise on dietary decisions and/or weight loss were desired. ‘Being accountable to someone else’ was considered to be a positive thing.

Overall, and importantly, respondents perceived ‘programs’ as consisting of short-term education by HCPs, whereas ‘support’ was regarded as daily interactions with friends and family.

Concepts and themes regarding diabetes management and support strategies that emerged from the patients’ descriptions informed the creation of quantitative survey questions and response options that reflected the patients’ experiences. These themes included, but were not limited to, weight loss as an area of concern and as a goal for T2DM programming; the importance of modes of support, such as printed materials, in T2DM education; sources of support, such as spouses, partners, family, and friends, in effectively managing T2DM; and how personal expectations/hopes for T2DM can align with goals of T2DM support programming.

### Quantitative survey process

#### Survey development

Based on the results from the qualitative interviews and literature review, a survey consisting of a maximum of 90 questions (accounting for branching and variable questions asked only in response to specific answers to prior questions) and including both closed (Likert-scale, multiple-choice, matrix, and numerical) and open (free-form text) response formats was created [see Additional file 2]. Patient-reported interview data were used to frame and develop questions for use in the quantitative survey. For example, descriptions of diabetes support programs obtained in response to questions from Part 3 of the qualitative survey [see Additional file 1] were used to construct the support program types mentioned in Sections IV, V, and VI of the quantitative survey [see Additional file 2] as well as to provide lists of commonly mentioned response options.

In the survey, respondents were asked to confirm the demographic information previously collected from their profiles by completing a basic demographics panel prior to addressing the main survey questions. Demographic data concerning sex, date of birth, ethnicity, race, educational level, and health-insurance type were collected. Location information, such as country and state, were re-collected from respondents at this point as well. In addition to the demographic covariates, the main survey was composed of seven domains which were framed from the patient perspective:

1. Overall quality of life – to understand all areas of health of the patient
2. Diabetes goals – to focus on what patients want from their disease control
3. Current and past programs – to assess program participation and preferences/dislikes
4. Preferences for self-support – to understand the types, sources, and formats of support that patients want when managing their diabetes
5. Support network – to give an insight into who (or what) comprises the patient’s current support network
6. Treatments and complications – to provide data on what patients have experienced
7. Comorbidities – to provide data on patient-identified additional conditions

An appended sub-survey queried patients on the following: their satisfaction with their health-care; difficulties managing symptoms; glycated hemoglobin A_1c_ (A1C), low-density lipoprotein, and blood pressure measurements; use of oral steroids; weight-loss goals; health literacy; and self-reported treatment adherence.

Branching within the survey was incorporated to address program characteristics, information covered, program length, and outcomes for respondents who participated in a program. Approximately 20 of the 90 questions were in free-form text format, which queried survey respondents on what ‘other’ types of programs, information, or diabetes support they participated in, wanted, or preferred.

#### Study population

The online survey was fielded during April to July 2014 to active PLM participants reporting T2DM as a condition on their profile, who also reported residing in the USA or did not specify their location and who were aged ≥ 18 years. The initial pool of invitations was limited to the most active patients (those who had logged in to the site in the past 90 days). A second pool of invitations was created for participants who had lower activity (at least one log-in during the past year).

#### Survey fielding

Patients fulfilling study inclusion criteria were emailed an invitation to participate in a custom survey. The survey fielding was conducted in two waves, a pilot survey and a full survey. The pilot survey was fielded to assess the interpretability of questions by a sample of 100 members with T2DM, and remained open for 2 weeks. The full survey was fielded to the larger T2DM population and remained open for a period of approximately 12 weeks. For both the pilot and full surveys, the invitation to participate appeared as a private message when a T2DM patient logged in to the site. The invitation included a link directing the patient to where the survey could be completed online. Users who did not complete surveys within 3 days were sent an email reminder to participate in the survey.

#### Statistical analyses

The population for the main analysis consisted of all confirmed US-based patient respondents who fulfilled the inclusion criteria. As there were no changes to the survey, responses obtained during the pilot survey were merged with full survey results prior to data analysis. To provide additional context on the PLM T2DM community, survey respondents were compared with nonrespondents with respect to their demographic characteristics, any listed (additional) conditions, and their PLM website participation as a preface to the main analyses. Before analyzing survey results, available profile data (such as location, primary condition, and zip code) were matched to survey responses by a unique user ID number. Zip codes were then grouped according to area type (urban, suburban, rural) using US Census data.

Descriptive statistics were used to describe patient characteristics by demographic covariates of interest, and summary statistics were used to tabulate frequencies and relative percents, such as patient use of different self-management support systems. *χ*^2^ statistics were used for categorical variables, and two-sample *t*-tests were used to compare groups for continuous covariates. Trend tests were used to test two-category predictor variables on ordinal-ordered outcomes, such as program participation and satisfaction with one’s health-care. Agreement between binary paired variables, such as past and current program participation, was assessed with the kappa statistic. Only *P*-values of ≤ 0.05 were reported, and all tests were two-tailed. Free-text or ‘other’ text response options were analyzed by summarizing key concepts by ‘theme’ and enumerating the mention of each theme. Quantitative analyses were conducted with SAS, Version 9.4 (Cary, NC, USA).

### Ethics, consent, and permissions

This study was approved by the Western Institutional Review Board on December 31, 2013. Materials sent to study participants such as invitation messages, together with the interview guide, research information, and participant consent were reviewed and approved by the Review Board. Using online means, the nature of the study was explained to potential participants prior to participation, and informed consent was obtained for participation in the study via an online informed consent form. Verbal consent was obtained for the audio recording of verbal interviews.

## Results

### Quantitative survey findings

#### Survey metrics

**Fig. 1:**
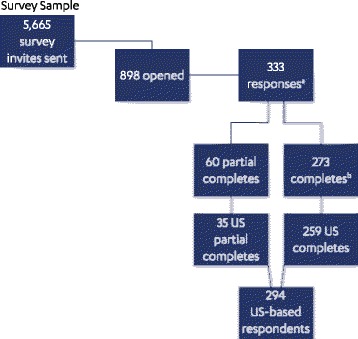
Flow diagram of study participants. ^a^Participation rate = 37.1 %; ^b^Completion rate = 30.4 %

A survey participant flow diagram is shown in Fig. 1. A total of 5665 PLM users who fulfilled the inclusion criteria were sent the survey invitation. Responses (including partial responses) were received from 294 confirmed US-based invitees, and these formed the total sample for analysis.

#### Survey sample characteristics

Table 1 shows the demographic characteristics of the PLM sample of adults with T2DM; the survey sample was mostly white (83.9 %) and female (64.3 %), with a mean age of 56.5 years.

**Table 1:** Demographic characteristics of the PLM T2DM population

Characteristic	PLM sample (*N* = 294)
Age, mean (±SD), years	56.5 (±10.6)
Sex, *n* (%)	
Male	105 (35.7 %)
Female	189 (64.3 %)
Race, *n* (%)	
White	245 (83.9 %)
Black/African-American	21 (7.2 %)
Mixed race	10 (3.4 %)
Asian	5 (1.7 %)
Native American	1 (1.0 %)
Prefer not to say	10 (3.4 %)
Ethnicity, *n* (%)	
Hispanic	10 (3.4 %)
Self-reported^a^ A1C, mean (±SD), %	7.04 (±1.6)
Educational level, *n* (%)	
High-school degree or less	51 (17.8 %)
Some college	129 (45.1 %)
College graduate	65 (22.7 %)
Postgraduate education	41 (14.3 %)

*A1C* glycated hemoglobin A_1c_, *SD* standard deviation; ^a^ *N* = 146

The demographic characteristics of respondents versus nonrespondents were first compared between the samples, and the populations were found to be generally similar. The majority of survey respondents and nonrespondents were white (83.9 and 82.3 %, respectively), aged 56 years on average (56.5 vs 55.5 years), and had some college education (45.1 % vs 46.7 %). Significantly more respondents than nonrespondents were insured via Medicare (35.9 % vs 25.3 %, respectively; *P* < 0.01) and slightly more were male (35.7 % vs 28.6 %; *P* = 0.03).

Survey respondents were assessed on their general health, quality of life, and diabetes goals. The mean A1C level of respondents (*n* = 146) was 7.04 %, showing that glycemic control was good in almost 50 % of patients who provided information. Overall, most respondents (65.2 %) rated their general health to be fair (33.8 %) or good (31.4 %), and about half reported little-to-no distress in feeling overwhelmed by the demands of living with diabetes (56.1 %) or feeling that they were often failing with their diabetes regimen (50.2 %). The majority of respondents (88 %) reported having ≥ 1 key comorbid condition in addition to their T2DM. Overweight/obesity (72.8 %), high blood pressure (64.3 %), high blood cholesterol (63.6 %), and depressive disorders (53.1 %) were the most commonly selected comorbid conditions.

#### Diabetes program utilization

**Fig. 2:**
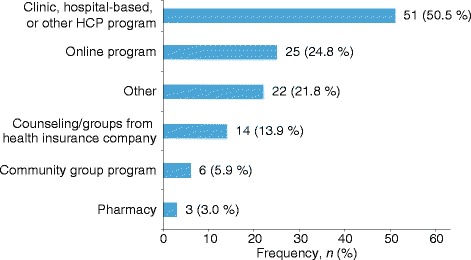
Program utilization among survey respondents currently participating in a form of support^a^ (*n* = 101). HCP, health-care professional. ^a^Respondents could have participated in more than one program

Figure 2 shows the current program utilization by survey respondents. Among the 285 respondents, nearly two-thirds reported not currently participating in any program or not having participated in any diabetes management programs in the past. Of those currently participating in a program, nearly two-thirds reported a positive change in their diabetes (Table 2) and more than 18 % believed that they were ‘doing much better.’

**Table 2:** Change in diabetes as a result of program participation

Question/statement	Frequency (*N* = 91)
Have you noticed any change in your diabetes as a result of participating in a program? *n* (%)	
I am doing much better	17 (18.7 %)
I am doing a little better	42 (46.2 %)
I haven’t noticed any change in my diabetes	28 (30.8 %)
I am doing a little worse	4 (4.4 %)
Change in diabetes as a result of program participation *n* (%)	
No change or doing worse	
Clinic, hospital-based, or other HCP program Counsel groups insurance Community group Pharmacy Online Other Total	14 (15.4 %) 3 (3.3 %) 1 (1.1 %) 0 (0.0 %) 9 (9.9 %) 5 (5.5 %) 32 (35.2 %)
Doing a little or a lot better	
Clinic, hospital-based, or other HCP program Counsel groups insurance Community group Pharmacy Online Other Total	23 (25.3 %) 8 (8.8 %) 3 (3.3 %) 1 (1.1 %) 10 (11.0 %) 14 (15.4 %) 59 (64.8 %)
How likely would you be to repeat this program in the future? *n* (%)	
Extremely likely	13 (14.3 %)
Likely	29 (31.9 %)
Neutral	30 (33.0 %)
Unlikely	6 (6.6 %)
Extremely unlikely	13 (14.3 %)

*HCP* health-care professional

A significant association was found when comparing past program participation to current program participation (*χ*^2^ = 13.04; *P* < 0.01). While the majority of respondents who had not participated in programs in the past were currently not participating in a program (73.9 %), those who currently participate in programs were more likely to have participated in a past program than those who are currently not participating in a program (47.8 % vs. 26.1 %, respectively). However, the fact that a little less than half (48 %) of past program participants go on to participate in a program again is reflected in the kappa statistic of 0.22, indicating only fair agreement between past and present program participation.

Among those currently participating in programs (*n* = 101), respondents most commonly participated in clinic, hospital-based, or other HCP programs (*n* = 51; 50.5 %), followed by online programs (*n* = 25; 24.8 %) and ‘other’ personalized programs (*n* = 22; 21.8 %) that they designed/implemented on their own (Fig. 2). ‘Other’ programs mentioned by participants included specific diet plans, combination diet/exercise/lifestyle approaches, individualized daily planning, online programs and online counseling, and bariatric bypass surgery.

**Fig. 3:**
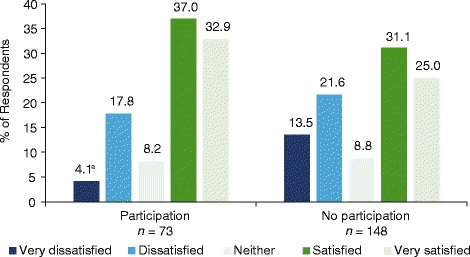
Overall satisfaction with health-care team in the past year by current program participation^a^. ^a^ *Z = −*2.28*; P* = 0.0022

Figure 3 shows the overall level of satisfaction of respondents with their health-care team in the past year according to current program participation. When questioned about their overall level of satisfaction with their health-care team in the past year, respondents who were currently participating in a program had significantly greater satisfaction with their health-care team during the past year than those who were not participating (*Z* = −2.28; *P* = 0.002 for trend). Dissatisfaction likewise appeared to be lower among current program participants.

#### Patient preferences for diabetes self-management programs and support

**Fig. 4:**
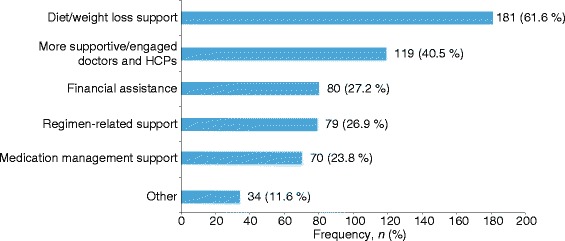
Types of support preferred by survey respondents. HCP, health-care professional

**Fig. 5:**
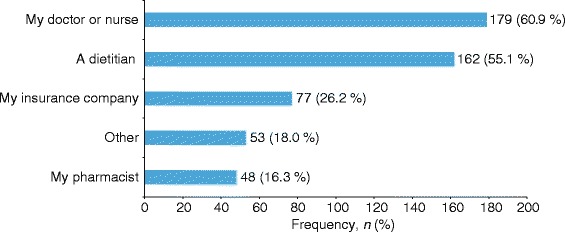
Sources of support preferred by survey respondents

Figure 4 gives an overview of the types of support preferred by survey respondents (*N* = 294). Overall, diet/weight loss support was the most preferred type of support, followed by more supportive/engaged doctors and other HCPs. Doctors or nurses (*n* = 179; 60.9 %) and dietitians (*n* = 162; 55.1 %) were the most preferred sources of diabetes support among the survey respondents (Fig. 5).

The most frequent goals set by survey respondents were ‘eat a better diet/manage food’ (*n* = 226, 76.9 %), ‘lose weight’ (*n* = 210, 71.4 %), ‘keep blood sugar levels stable’ (*n* = 210, 71.4 %), and ‘keep A1C value at a certain level’ (*n* = 199, 67.7 %), followed by ‘do regular physical activity’ (*n* = 153, 52.0 %) and ‘manage stress better’ (*n* = 119, 40.5 %). Only 20 respondents reported having set no goals for themselves at all (6.8 %).

**Fig. 6:**
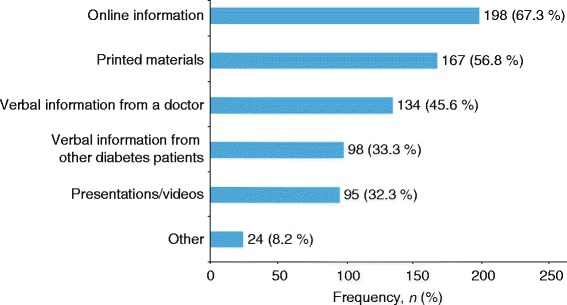
Format of support most preferred by respondents^a^ (*n* = 294). ^a^Participants could select more than one form of support

When queried about the formats of support information they most preferred, respondents most frequently preferred online information, followed by print materials and verbal information from a doctor (Fig. 6). Of the 184 respondents who reported receiving diabetes support from other people, 76.6 % received support exclusively from doctors or nurses and only 23.4 % of respondents received diabetes support from individuals other than doctors or nurses (family, friends, and others).

## Discussion

Diabetes management programs have positive effects on the health of patients with T2DM; however, due to the heterogeneity of program designs and the outcomes studied, the most effective components of diabetes management programs are not well understood [11, 14]. This study is the first to attempt to elucidate the types of diabetes management programs and the features of those programs that are valued from a patient perspective. It does so by gathering the opinions of patients with T2DM.

Recent positions of the ADA and EASD point to individualized and tailored care [4]. As such, this investigation of the goals set by survey respondents with respect to their own diabetes management gave valuable insights into what the participant actually considers important. The most frequently selected goals set by our survey respondents were better diet (76.9 %) and weight loss (71.4 %). Weight loss has been shown to have the most positive effect on treatment satisfaction and quality of life in patients with T2DM [8, 15–18]. As individuals are more likely to adhere to treatment regimens that offer benefits from a patient’s perspective, by including weight loss as a major component of patient support systems, both participation in and the effectiveness of these programs may increase.

In this analysis, patients with T2DM reported greater satisfaction with the care received from their health-care team in the past year if they were currently participating in a support program. This is noteworthy, and there are multiple implications inherent in this association. Either those who are more satisfied with their health-care team tend to participate in programs, program participation makes participants more cognizant/appreciative of their HCP’s efforts, or program participation in itself leads to better health-care and, subsequently, more satisfaction with it on the part of patients. Compared with nonparticipants, a patient joining a support program may be more actively involved in his/her own care (i.e., increased patient engagement) and more vocal with their provider about the care they need. This, in turn, may allow providers to meet patients’ needs more closely, leading to program participants being more satisfied with the health-care received.

An important point emerged from the study concerning the interaction between preferred sources and preferred formats of support. Although respondents expressed the desire for more supportive/engaged doctors and other HCPs, and found doctors, nurses, and dietitians to be the most preferred sources of diabetes support, there was a clear preference for online and printed materials as support format above verbal information. This identifies an interesting need that could potentially be addressed by providing HCPs with useful online educational tools and by giving them access to a library of practical printed materials. These support formats could be used by HCPs as a practical means to reach out to and engage with their patients in the manner that many patients have expressed as their preference. The findings suggest that being specifically referred to online or printed materials by their doctors would be viewed positively and could even increase the perception of doctors as being supportive and engaged. There is a clear need for HCPs to be aware of patients’ needs for multifaceted T2DM support, as in many cases patients are not receiving the support they need from any other source.

The current study represents, to the authors’ knowledge, the first investigation of patients’ own preferences for support in the context of diabetes management programs. The information was obtained directly from a cross-section of patients with T2DM in a real-world setting. Although this study provides a snapshot of a situation at a given time, the results present the voices of patients who are at various points along the T2DM continuum. The study used a mixed-methods approach, combining both qualitative and quantitative research. The qualitative information gathered from a small number of patients with T2DM, representing the spectrum of patient demographics, allowed for a very clear and directed survey to be designed to maximize the effectiveness of the study. An additional strength of this study was the breadth and depth of the interviews conducted, which allowed the collection of a rich and comprehensive data set that can contribute towards the understanding of patient preferences and motivation for management of T2DM.

While the mixed-methods approach of using the results of a qualitative survey to design a quantitative survey was a strength of this study, the lack of using a validated questionnaire assessing program participation and patient engagement was a limitation. It should, however, be noted that the process by which validated questionnaires are created was operationalized in the current study: a patient focus group was used for concept elicitation, and themes arising from the focus group were used in the development of survey items, domains, and response options. The survey also included a considerable amount of ‘free-text responses’ where patients could respond in their own words, allowing for further thematic exploration.

Another limitation of this study was sample generalizability. The majority of respondents were white middle-aged, and had a higher educational level than the general population with T2DM, which hampered our ability to generalize these results to T2DM patients at large. There were limited distributions of age, education, and minority status, all potential risk factors for T2DM which lend themselves to worse diabetes outcomes. The PLM population is skewed towards a more female, educated, and engaged group of patients, reflecting the patient population who regularly use health-based Internet sites [19, 20]. Since study respondents were active users of PLM, the sample was reflective of a more highly engaged group of patients who actively seek information, particularly online materials, and who are likely to be more proactively involved in the management of their own condition than many other patients with T2DM. This higher engagement is also reflected in the mean A1C level (7.04 %) reported by the PLM sample, which is largely considered well-controlled.

Nonetheless, respondents’ self-reported participation in self-care management programs was low, reflecting trends seen in other T2DM populations [21, 22]. In fact, the percentages cited here are likely at best an underestimate of nonparticipation in these programs in the T2DM population as a whole. Engaged patients are most often vocal about what does and does not work in T2DM self-management and support programming, and are most likely to share their hopes and goals for condition management. Learning about program participation in an engaged population of real-world patients allows researchers the unique opportunity to begin to fill the gaps in what little is known about patient preferences for and desires of T2DM care [23]. It is also arguable that T2DM self-care management and programs are, in fact, more effective when based around a group of patients who are more engaged with their health and who are active participants in their own care.

In the future, it will be important to investigate patient perspectives regarding the impact of program participation or nonparticipation. It will also be informative to understand in greater detail the types and features of programs that gain the attention of patients and appear to be sufficiently attractive for them to take part in. Longitudinal studies could directly follow the effects of program participation on T2DM outcomes, such as A1C levels, weight loss, and lipid control. Furthermore, there is a need to explore physician perspectives and those of other HCPs, in addition to the preferences of patients, because challenges facing HCPs are also important for the development of successful diabetes management programs. Such studies, which aim to understand patient and physician preferences for diabetes support and management programs, will ultimately inform future recommendations for HCPs and could potentially result in improvements in the overall care of patients with T2DM.

## Conclusions

This study, based on a real-world sample of patients with T2DM, revealed that the majority of patients were not participating in any program. Among those using a T2DM self-management program, respondents most frequently used clinic, hospital-based, or other HCP programs. Diet and weight loss were the most important components of diabetes management and support programs preferred by patients, followed by supportive/engaged doctors and other HCPs.

The results of this study may help to establish a foundation that can advise on the design of future T2DM management support, leading to more effective programs that will be modifiable within the context of the individualization of care that is currently recommended by the ADA and EASD.

## Abbreviations

A1C, glycated hemoglobin A_1c_; ADA, American Diabetes Association; EASD, European Association for the Study of Diabetes; HCP, health-care professional; PCORI, Patient-Centered Outcomes Research Institute; PLM, PatientsLikeMe®; PPACA, Patient Protection and Affordable Care Act; T2DM, type 2 diabetes mellitus

## Additional files

Additional file 1: Qualitative interview process. Full description of the qualitative interview process, including the semi-structured interview guide, is provided in Additional file 1. (PDF 337 KB) Additional file 2: Quantitative survey. Structured survey questions are provided in Additional file 2. (PDF 500 KB)
