# Supplementary material for: Understanding preferences for type 2 diabetes mellitus self-management support through a patient-centered approach: a 2-phase mixed-methods study
Source: BMC Endocr Disord. 2016 Jul 18;16:41. doi: 10.1186/s12902-016-0122-x (PMC4950768; doi:10.1186/s12902-016-0122-x)
Supplement: Additional file 1: — Qualitative interview process. Full description of the qualitative interview process, including the semi-structured interview guide, is provided in Additional file 1. (PDF 337 KB) [file 12902_2016_122_MOESM1_ESM.pdf]

Additional file 1:

Qualitative interview process

Study population

Individuals who were actively participating in discussions on the PLM website (multiple login sessions on the PLM website including activity in the diabetes forums) and reported T2DM on their patient profiles were recruited for qualitative telephone interviews. We recruited ten individuals representing diverse age, race, and educational strata. Patients were invited by private message via the PLM site to participate in a 1-hour telephone interview. Those who met the inclusion criteria (stated US resident, aged ≥ 18 years, reporting T2DM on their profile) were sent a research information form and provided verbal consent for their interviews to be recorded. The invitation messaging, interview guide, and research information and consent were reviewed and approved by the Western Institutional Review Board on December 31, 2013.

Procedures

Interviews

Individual interviews were conducted in January 2014 and were based on the principles of grounded theory, which seeks to produce spontaneously elicited, rich descriptions of the issues and supports used by patients with diabetes [1]. With this approach, concepts emerge from patients' input, allowing the voice of the patient to be heard rather than applying an *a priori* theoretical model or constructs to interpret the data [2].

[1] Glaser, BG, Strauss, AL. The discovery of grounded theory: strategies for qualitative research. Chicago: Aldine; 1967.
[2] Charmaz, K. Grounded theory. In: Hesse-Biber SN, Leavy P, editors. Approaches to qualitative research. New York: Oxford University Press; 2004. p. 496-521.

An open-ended, semi-structured interview guide was created that included specific topic areas of interest and domains identified in the literature. These included experiences with controlling blood glucose levels, learning about diabetes and its complications, managing symptoms and treatment regimens, types of programs and support systems used, different people who are involved with managing diabetes, and satisfaction with program elements. The topics directed the interview through three parts: background on the patient's experiences with diabetes; issues and concerns in managing their condition; and self-management support strategies and aids. A typical open-ended question for the interview guide was: 'What is important for you to manage day-to-day with your diabetes?' All interviews were conducted by a single interviewer, one of the authors (MF-R), a clinician with training and experience conducting qualitative interviews. Interviews typically took approximately 1 hour, were audio recorded, and notes were taken throughout.

Qualitative data analysis (data analysis)

Concepts and themes regarding diabetes management and support strategies emerged from the patients' descriptions during the interviews. These included: identification of the most important symptoms patients felt they needed to control; who helps patients manage their diabetes; patients' perceived need for more diet and weight loss assistance; patients' goals and expectations regarding disease management; and specific patient preferences for support information that could be used to individualize their diabetes control plans. These concepts informed the creation of specific survey questions and response options that reflected the patients' experiences. There were also two concepts elicited from patients that informed specific sections of the final survey: the importance of encouragement from others in adhering to plans, and the difference between 'programs' (short-term education provided by HCPs) and 'support' (daily interactions with family, friends, and others).

Semi-structured interview guide

Type 2 diabetes mellitus self-management support systems - Qualitative interviews

Introductory invitation message to selected patients:

> My name is **Marcy Fitz-Randolph** and I work as a researcher at *PatientsLikeMe*. We want to learn more about how patients with diabetes take care of themselves and their disease. Would you be willing to speak with me about your experiences with managing diabetes? We'd meet for about an hour online via Skype or Google Hangout, or by telephone if that would be easier. We would give you a $25 honorarium as a thank you for your time.

In the process of scheduling the interview, the patient's age, gender, and time of diagnosis will be confirmed (against known profile data).

At the time of the interview, the interviewer will ascertain through conversation that the patient is the person who corresponds to the PatientsLikeMe (PLM) profile targeted. If there are any questions, the interviewer will ask directly if the patient is the person described in the PLM profile.

If the interview is ended prior to the final questions, either by the patient or through technical issues, partial information will be retained for analysis, without the confirming demographic questions asked at the end of the interview.

Interview guide

Section 1 – Overview

<u>Objective</u>

Review the purpose of the study and confidentiality arrangements, and ask patient's permission to audio-record the interview to augment handwritten notes.

Interview introduction:

Thank the participant for agreeing to participate in this interview.

Introduce yourself as working on behalf of PatientsLikeMe on a research project with an external supporter.

Explain this particular project:

Example:

"The aim of this interview is to find out about your experiences with getting support or help with you taking care of your diabetes."

Reassure patients of confidentiality:

Example:

"Your name and contact information will remain with *PatientsLikeMe* and will only be accessible to people directly involved with this project. Any information you provide will be aggregated with other patient interviews, and *PatientsLikeMe* will protect your privacy and will not share your name or any other information that could identify you. The notes from this interview will be shared with the supporter of this study, but your name or any information that identifies you will be removed first."

Discuss audio-recording of the discussion:

Example:

“I would like to audio-record today’s interview to enable me to pay careful attention to what you say and to make certain we accurately capture the information that you provide to us during the interview. I will review the recording after the interview to finish my notes, then erase the recording.

“The recording will not be shared with our supporter. However, a written version of the notes from this interview will be prepared that removes any information that would identify you personally.

“Please try to speak relatively loudly so that your comments can be heard and are clear on the recording.

“Please be honest and open in your comments and don’t be afraid to voice any of your opinions.

“You may stop the interview at any point you feel the need to, and you may decline to answer any question.”

Wrap up the introduction:

Example:

“The interview today will take approximately one hour.”

“Do you have any questions at this point?”

NOTE TO INTERVIEWER:

- It is important not to lead respondents, as we want their open responses. However, please probe where appropriate and in a dynamic fashion. It is important for all of the main areas of the interview guide to be covered; however, **if a topic comes up earlier, you may discuss it at that time**. **The interview guide is not a script**.
- If the respondent directs any questions of a medical nature toward you, please explain that you are not a medical professional and that the patient should direct any medical questions to their physician.
- Expect the respondent to take a significant amount of time to respond to each question. Allow them enough time to answer.

Section 2 – Experience of diabetes and self-care support

<u>Objective</u>

The objectives for this interview are to explore patients' experiences with self-care support systems and modes of delivery for patients with type 2 diabetes mellitus.

NOTE TO INTERVIEWER:

Please remember to allow patients to respond spontaneously to the following questions. If patients mention something in relation to their symptoms or condition, please encourage the patient to talk more about the issues. **PROBES** have been included; use only where patients have not spontaneously mentioned the relevant issues.

"During this interview we will be talking about your diabetes. I would like to understand your experiences with diabetes and the ways you manage it. I'd also like to understand where and how you get support for taking care of yourself with diabetes."

<u>Part 1 – Introduction</u>

"What do you think about most when you think about your diabetes?"

"What bothers you the most about having diabetes?"

"How can you tell you are having problems with your diabetes?"

"What types of support would you like to receive that would make your life with diabetes easier? What would you like to have more of?"

[For each symptom, problem or comorbidity, ask:

- What is it like to have [symptom]?
- How does [symptom] impact you? Can you give me an example?
- How do you manage [symptom]? Can you give me an example?
  {make note for Part 2}
- How easy or hard is it for you to manage [symptom]?]

Part 2 – Managing diabetes

"We've talked some about managing your diabetes. Do you have any specific goals for yourself with managing your diabetes?"

"What is important for you to manage day-to-day with your diabetes? Why?"

> **PROBES**: *medications, nutrition, physical activity, low blood sugar/hypoglycemia, weight management, checking your glucose*

"What do you expect will happen to you with your diabetes by taking care of these things? What do you hope will happen?"

> **PROBES**: *immediate effects; short-term, long-term outcomes*

"What things related to your diabetes do you not manage every day? Why?"

[For items mentioned as managed but not daily, ask:

- How often do you need to think about [thing]? Can you give me an example?
- When do you think about managing [thing]?
- Is it difficult to manage [thing]?]

PROBE: [For items mentioned as not being managed at all, ask:

- "Is [thing] something you worry about? Why/why not?"]

"Are there other people who help you manage your diabetes, like your family or your doctor?"

[For each person mentioned, ask:

- Who helps you?
- How do they help you? Can you give me an example?
- How do you feel about the help?]

"Are there things related to your diabetes that other people might help you manage, but they are not?"

[For each item mentioned, ask:

- Who would that be?
- How would they help you? Can you give me an example?
- How do you feel about not having that help?]

Part 3 – Self-management supports

"Some people with diabetes have support systems or programs to help manage their diabetes. Are you familiar with any diabetes support programs or things to help you manage your diabetes?"

[For any supports given, ask:

- Have you tried that?
    - How long did you try it for (to get at drop-off in participation)
- What was it like?
- How was it offered (modality)?
- Where did you get it? (physician, other patients, insurance, etc.)

PROBES: *group class, individual class, online, printed material, mobile apps, exercise class, others*

- What information was covered / what did it help you do? Can you give me an example?
- How much effort was it to do?
- How did you stay motivated to do it? Can you give me an example?
- What did you like/not like about it? Can you give me an example?
- Did your diabetes change while you were doing it? Can you give me an example?
- Why are you still doing it? / Why did you stop doing it?]

"What other programs or things do you know of to help people manage their diabetes? What do you wish existed?"

[For other programs not tried or wished for, ask:

- What information was covered? Can you give me an example?
- What did you like/not like about it?
- Why did you not try it?]

"What kind of help do you like/prefer with managing your diabetes?"

[For any preferences given, ask:

- Why do you like that?
- Why is that better for you?
- How do you think it helps/would help you?]

"What kind of help do you dislike with managing your diabetes?"

[For any dislikes given, ask:

- Why do you not like that?
- Why does that not work for you?]

"What advice would you give someone with diabetes about support or programs for taking care of their diabetes? What worked for you that might work for someone else?"

"That finishes my questions for you about your diabetes and how you manage it. Thank you for all the information. Is there anything else we should know, or that you want to tell me about managing your diabetes?"

TURN OFF RECORDING

"I have a few last questions I would like to ask you to help group your answers with other patients like you. Would you mind giving me your…"

- Race
- Hispanic/not Hispanic
- Highest grade completed/educational level
- ZIP code
- Insurance status
- Employment status (full-time, part-time, student, retired, unable to work/disabled)
- Family income group
