# Supplementary material for: Understanding preferences for type 2 diabetes mellitus self-management support through a patient-centered approach: a 2-phase mixed-methods study
Source: BMC Endocr Disord. 2016 Jul 18;16:41. doi: 10.1186/s12902-016-0122-x (PMC4950768; doi:10.1186/s12902-016-0122-x)
Supplement: Additional file 2: — Quantitative survey. Structured survey questions are provided in Additional file 2. (PDF 500 KB) [file 12902_2016_122_MOESM2_ESM.pdf]

Additional file 2:

Quantitative Survey

STRUCTURED DIABETES SUPPORT SURVEY

Section I: Basic Information

Birth Date (Age)
Gender
Country
State (if US)
Health insurance
Ethnicity
Race
Educational Level

Section II: Overall, QoL

What is your work status? Please check all that apply.

Employed full-time (40 or more hours per week)
Employed part-time (less than 40 hours per week)
Self-employed
Unemployed
Homemaker
Student
Retired
Medically unable to work / disabled

Would you say your general health is:

Excellent
Very good
Good
Fair
Poor
Don't know/not sure

In the past month, how much has your diabetes impacted your daily activities? (multiple choice)

Not at all
A little
Some
A lot
Extremely

Section III: Symptoms & Goals

Please indicate the degree to which the following feelings may have distressed you during the past month by marking the appropriate number.
[matrix question; response columns 1–5]

1 = no distress
2 = a little distress
3 = moderate distress
4 = significant distress
5 = extreme distress

[matrix question rows]
Feeling overwhelmed by the demands of living with diabetes.
Feeling that I am often failing with my diabetes regimen.

What types of personal goals have you set for yourself with respect to your diabetes management?
[multiple choice; check all that apply]

Did not set any specific goals
Keep my A1C value at a certain level
Do regular physical activity
Keep my blood sugar levels stable
Lose weight
Eat a better diet/manage my food
Manage stress better
Other: [branch to free-text response box]

What do you ***most*** hope to achieve in terms of managing your diabetes by sticking to your goals?
[multiple choice]

Reducing the amount of medication I take
Eliminating the need for medication for diabetes
Avoiding diabetic complications (such as heart disease, stroke, kidney failure, nerve pain/tingling/numbness, foot ulcers, retinal eye disease, and others)
Preventing symptoms from getting worse
Other [branch to free-text response box]

Section IV: Current self-care/support programs

Now we'd like to ask you about experiences you've had with diabetes support programs or self-care management programs. These include programs offered through your insurance plan, pharmacies, community-based programs, web-based programs or applications, or other formal or informal diabetes support groups.

Which of the following methods do you **currently** use to manage your diabetes?
[multiple choice, check all that apply]

I am in a program run by a clinic, hospital, or other health care practitioner
I am in counseling/groups from my health insurance company
I am in a community group program

I am in a program run by my pharmacy
I am in an online program
Other [branch to free-text response box]
None of the above

[if yes] Thinking about the **one** current program you are **most satisfied** with, please answer the following questions.

Which current diabetes program are you thinking about when answering the following questions?
[multiple choice]
A program run by a clinic, hospital, or other health care practitioner
Counseling/groups from my insurance company
A community group program
A program run by my pharmacy
An online program
Another type of program

How long have you participated in this program?
[multiple choice]
Less than a week
One week to a month
More than 1 month
Between 1 month and 6 months
More than 6 months but less than one year
A year or more

How many days in the past three months have you participated in the program?
[number 0–92]

What information is covered in this diabetes program?
[multiple choice, check all that apply]
Controlling blood sugar levels
Self-monitoring strategies and tools (blood glucose and other tests such as A1C)
Medication assistance (for example, help paying for prescriptions)
Dealing with blood pressure control, cholesterol levels, eye and kidney health
Diet support or healthy eating (including dietary consultations)
Exercise program (either in gym or other)
Weight loss strategies
Other [branch to free-text response box]

How motivating is this program in helping you manage your diabetes?
[multiple choice]
Not at all motivating
A little motivating
Somewhat motivating

Motivating
Very motivating

How much effort does this program require on your behalf in order to be effective?
[multiple choice]
No effort
A little effort
Moderate effort
A lot of effort
Extreme effort

Have you noticed any change in your diabetes as a result of participating in this program?
[multiple choice]
I am doing much better
I am doing a little better
I haven't noticed any change in my diabetes
I am doing a little worse
I am doing much worse

How likely would you be to repeat this program in the future?
[multiple choice]
Extremely unlikely
Unlikely
Neutral
Likely
Extremely likely

Do you currently use any of the following methods to manage your diabetes?
[multiple choice, check all that apply]
I use websites to help me monitor my diabetes by myself
I use health apps designed to help me monitor my diabetes by myself
I regularly read material I find online to educate myself
I regularly read material from places other than online to educate myself
I have informal support and discussions with friends, family, or other diabetes patients
Other [branch to free-text response box]
None of the above

Section V: Past experiences with self-care/support systems

Are there any diabetes support programs that you participated in before that you are no longer doing?
[multiple choice, check all that apply]
I was in a program run by a clinic, hospital, or other healthcare practitioner
I was in counseling/groups from my health insurance company
I was in a community group program

I was in a program run by my pharmacy
I was in an online program
I was in another type of program [branch to free-text response box]
I have never been in any programs in the past

[If any but "I have never been in any programs in the past"]
Thinking about **one** program you participated in before and were the **most satisfied** with, but are not doing currently, please answer the following questions.

Which prior diabetes program are you thinking about when answering the following questions?
[multiple choice]
A program run by a clinic, hospital, or other healthcare practitioner
Counseling/groups from my insurance company
A community group program
A program run by my pharmacy
An online program
Another type of program

How long did you participate in this program?
[multiple choice]
Less than a week
One week to a month
More than 1 month
Between 1 month and 6 months
More than 6 months but less than one year
A year or more

What information was covered in this diabetes program?
[multiple choice, check all that apply]
Controlling blood sugar levels
Self-monitoring strategies (blood glucose and other tests such as A1C)
Medication assistance (for example, help paying for prescriptions)
Dealing with blood pressure control, cholesterol levels, eye and kidney health
Diet support or healthy eating (including dietary consultations)
Exercise program (either in gym or other)
Weight loss strategies
Other [branch to free-text response box]

How motivating was this program in helping you manage your diabetes?
[multiple choice]
Not at all motivating
A little motivating
Somewhat motivating
Motivating
Very motivating

How much effort did this program require on your behalf in order to be effective?
[multiple choice]

- No effort
- A little effort
- Moderate effort
- A lot of effort
- Extreme effort

Did you notice a change on your diabetes as a result of participating in this program?
[multiple choice]

- I did much better
- I did a little better
- I hadn't noticed any change in my diabetes
- I did a little worse
- I did much worse

Section VI: Preferences for self-support

What types of formal or informal support would you like to receive with your diabetes?
[multiple choice; check all that apply]

- More supportive/engaged doctors and health care providers
- Medication management support
- Diet/weight loss support
- Regimen-related support (blood sugar testing, daily scheduling, renewing and picking up medications, making appointments, etc.)
- Financial assistance
- Other [branch to free-text response box]

From what sources would you like to receive diabetes support?
[multiple choice; check all that apply]

- My doctor or nurse
- My insurance company
- My pharmacist
- A dietitian
- Other [branch to free-text response box]

In what format do you like to receive diabetes support?
[multiple choice; check all that apply]

- Printed materials
- Online information
- Verbal information from my doctor
- Verbal information from other diabetes patients
- Presentations/videos
- Other [branch to free-text response box]

Section VII: Support Network: caregivers/family/other people

Are there other people who currently help you to support your diabetes care, like family members, friends, or your doctor?

Yes
No

[If yes] I receive diabetes help, advice, management from:
[multiple choice; check all that apply]

My family
My friends
My doctor
My nurse
My pharmacist
My diabetes coaches or educators
Other diabetes patients
Other [branch to free-text response]

For each person who supports you, please check which types of support or information they currently give you. If you do not get support from a particular person, please check "Does Not Apply":
[Matrix question; rows are people from above question, responses are below]

[Matrix rows:]
My family
My friends
My doctor
My nurse
My pharmacist
My diabetes coaches or educators
Other diabetes patients

[Matrix response columns:]
Diabetes information
Diet support or healthy eating
Help with monitoring blood sugars
Exercise
Weight loss
Medication assistance
Helping to manage appointments
Social support
Encouragement
Enhancing quality of life
Does Not Apply

Section VIII: Treatments & Complications

Are you currently taking any medications to treat your diabetes?

[multiple choice: yes; no; don't know]

[If yes] What medications are you taking for your diabetes?
[multiple choice columns: Yes, No, Don't know]
[multiple choice rows: 8 categories below (table for ease of reading grouping]

Insulin
Sulfonylureas, such as:<br>• glimepiride (Amaryl)<br>• glipizide (Glucotrol)<br>• glyburide (Diabeta, Glynase)<br>• gliclazide (Glucozide, Diaglucide, others)<br>• nateglinide (Starlix)<br>• repaglinide (Prandin)<br>• **<u>or combinations</u>** with these
Biguanides, such as:<br>• metformin (Glucophase, Glumetza, others)
Thiazolidinediones, such as:<br>• pioglitazone (Actos)<br>• rosiglitazone (Avandia)<br>• **<u>or combinations</u>** with these
DPP-4 inhibitors such as:<br>• sitagliptin (Januvia)<br>• saxagliptin (Onglyza)<br>• linagliptin (Tradjenta)<br>• alogliptin (Nesina)<br>• **<u>or combinations</u>** with these
α-glucosidase inhibitors, such as:<br>• acarbose (Precose)<br>• miglitol (Glyset)
GLP-1 analogs, such as:<br>• exenatide (Byetta, Bydureon)<br>• liraglutide (Victoza)
Sodium-glucose cotransporter 2 inhibitors, such as:<br>• canagliflozin (Invokana)<br>• dapagliflozin (Farxiga)

Have you ever been told by a doctor, nurse, or other health professional that you have any of the conditions described below?[1] [matrix question: yes; no; don't know for each]:

- High blood pressure?
- High blood cholesterol?

[1] Common T2DM comorbidities from literature:
http://www.physiciansweekly.com/diabetes comorbidities-management/;
http://care.diabetesjournals.org/content/29/3/725.long

- A depressive disorder, including depression, major depression, dysthymia, or minor depression?
- A heart attack, also called a myocardial infarction?
- Angina or coronary heart disease?
- A stroke?
- Kidney disease because of your diabetes? (NOT kidney stones, bladder infection or leaking urine)
- Diabetes has affected your eyes or that you had retinopathy?
- Diabetes has affected your nerves/nervous system or that you have neuropathy?
- Sores or irritations on your feet that took more than four weeks to heal?
- Overweight/obesity?

Thanks for completing the first part of the survey! If you have another 10 minutes, we've got a few more questions to learn even more about your experiences.

Would you like to complete some more additional questions?
Yes [continue to optional questions below]
No {branch to final question:
Do you have any final comments about support systems and programs for diabetes? [free-text]
Do you have any final comments about this survey? [free-text]

Section IX OPT: Additional Questions (add-ons)

SECTION OPT1: Healthcare

The following questions ask about all your diabetes-specific health care. Include all the providers you saw for health care in the past year.

How satisfied are you with the help you got from your health care team (i.e. doctors, nurses, diabetes educator) to manage your care, tests, or treatment for your diabetes among these different providers in the past year?
[multiple choice]
Very dissatisfied
Somewhat dissatisfied
Neither
Somewhat satisfied
Very satisfied

[*If answered very dissatisfied, somewhat dissatisfied, neither in above*]
In your opinion, how could your experience from your health care team be improved? What types of support would be helpful?
[open text box]

[*If answered somewhat satisfied, very satisfied in above*]

In your opinion, what types of support did you get from your health care team that made you satisfied? [open text box]

SECTION OPT2: Diabetes Lab and Testing

How difficult is it for you to manage **each** of the following symptoms? If you do not have a particular symptom or problem, please check "Does not apply."
[matrix question; responses *not difficult/a little difficult/somewhat difficult/very difficult/does not apply*]

High blood sugar levels
Low blood sugar levels
Fatigue or tiredness
Weight changes (gain or loss)
Neuropathy (pain, numbness, tingling in feet or hands)

When was your last A1C test done?

Within the past month
Between one and three months ago
Between three and six months ago
Between six months and a year ago
More than a year ago
Don't know/don't recall

[If yes to any within the past year] My last A1C test result was (numeric range, 0–20)

Do you know your current blood pressure?

Yes
No

[If yes] Blood pressure readings are usually recorded with two numbers, [systolic] / [diastolic]. For example, 120/80.
My systolic (top number) blood pressure is: (numeric range, 30–300)
My diastolic (bottom number) blood pressure is: (numeric range, 30–300)

Do you know your current LDL-cholesterol (low-density lipoprotein) level?

Yes
No

[If yes] What was your last LDL-C? (numeric range, 0–400)

Do you know your current HDL-cholesterol (high-density lipoprotein) level?

Yes
No

[If yes] What was your last HDL-C? (numeric range, 0–400)

What is your current weight, in pounds?
[numeric response 60–600]

About how tall are you without shoes?
[multiple choice, 3'6" to 7'7"]

SECTION OPT3: Other

Have you ever taken oral steroid medications (for example, prednisone)?
Yes
No
Don't know/do not recall

[If yes] How often have you taken oral steroid medications?
[multiple choice]
Daily
Several times per week
Weekly
Every few weeks
Every few months
Less often than every six months
One time

[If response to earlier question about personal goals included "lose weight"]: Earlier, you mentioned that a personal goal you had was to lose weight. How many pounds do you feel you need to lose?
[numeric range, 0–300]

[If response to earlier question about personal goals included "lose weight"]: Who helped set your weight loss goal(s) with you?
[multiple choice]
I set this goal myself
My doctor/HCP
My spouse
My friend
Other [branch to free-text response box]

How confident are you filling out medical forms by yourself?[2]
Extremely
Quite a bit

[2] Al Sayah F, Majumar SR, Williams B, Robertson S, Johnson JA. Health Literacy and Health Outcomes in Diabetes: A Systematic Review. 2012; J Gen Intern Med 28(3):444–52; Chew LD, Griffin JM, Partin MR, Noorbaloochi S, Grill JP, Snyder A. Validation of Screening Questions for Limited Health Literacy in a Large VA Outpatient Population 2008; J Gen Intern Med 23(5):561–6.

Somewhat
A little
Not at all

How often do you follow your medical treatment plan (medications, measuring blood sugar levels, etc.)?
[multiple choice]
Never
Rarely
Sometimes
Often
All of the Time

Section X: Final Comments Question

Do you have any final comments about support systems and programs for diabetes? [free-text]

Do you have any final comments about this survey? [free-text]
